# Long non-coding RNAs as the critical factors during tumor progressions among Iranian population: an overview

**DOI:** 10.1186/s13578-020-0373-0

**Published:** 2020-01-14

**Authors:** Zahra Rahmani, Majid Mojarrad, Meysam Moghbeli

**Affiliations:** 1 Department of Medical Genetics, Golestan University of Medical Sciences, Gorgan, Iran; 2 Department of Medical Genetics and Molecular Medicine, School of Medicine, Mashhad University of Medical Sciences, Mashhad, Iran

**Keywords:** LncRNA, Diagnostic panel, Early detection, Cancer, Iran

## Abstract

**Background:**

Cancer is associated with various genetic and environmental risk factors. Beside the mutations or aberrant expression of protein-coding genes, the genetic deregulation of non-coding RNAs has also an important role during tumor progression and metastasis. Long non-coding RNAs (lncRNAs) are a class of ncRNAs larger than 200 nucleotides that may function as tumor-suppressor or oncogene.

**Main body:**

There is a raising trend of cancer incidence among Iranian population during the last decades. Therefore, it is required to prepare a general population specific panel of genetic markers for the early detection of cancer in this population. The tissue-specific expression characteristics and high stability in body fluids highlight the lncRNAs as efficient diagnostic and prognostic noninvasive biomarkers in cancer. In present review we summarized all of the lncRNAs which have been reported until now in different tumors among Iranian patients.

**Conclusions:**

This review paves the way of introducing a population based noninvasive diagnostic panel of lncRNAs for the early detection of tumor cells among Iranian population.

## Background

**Fig. 1:**
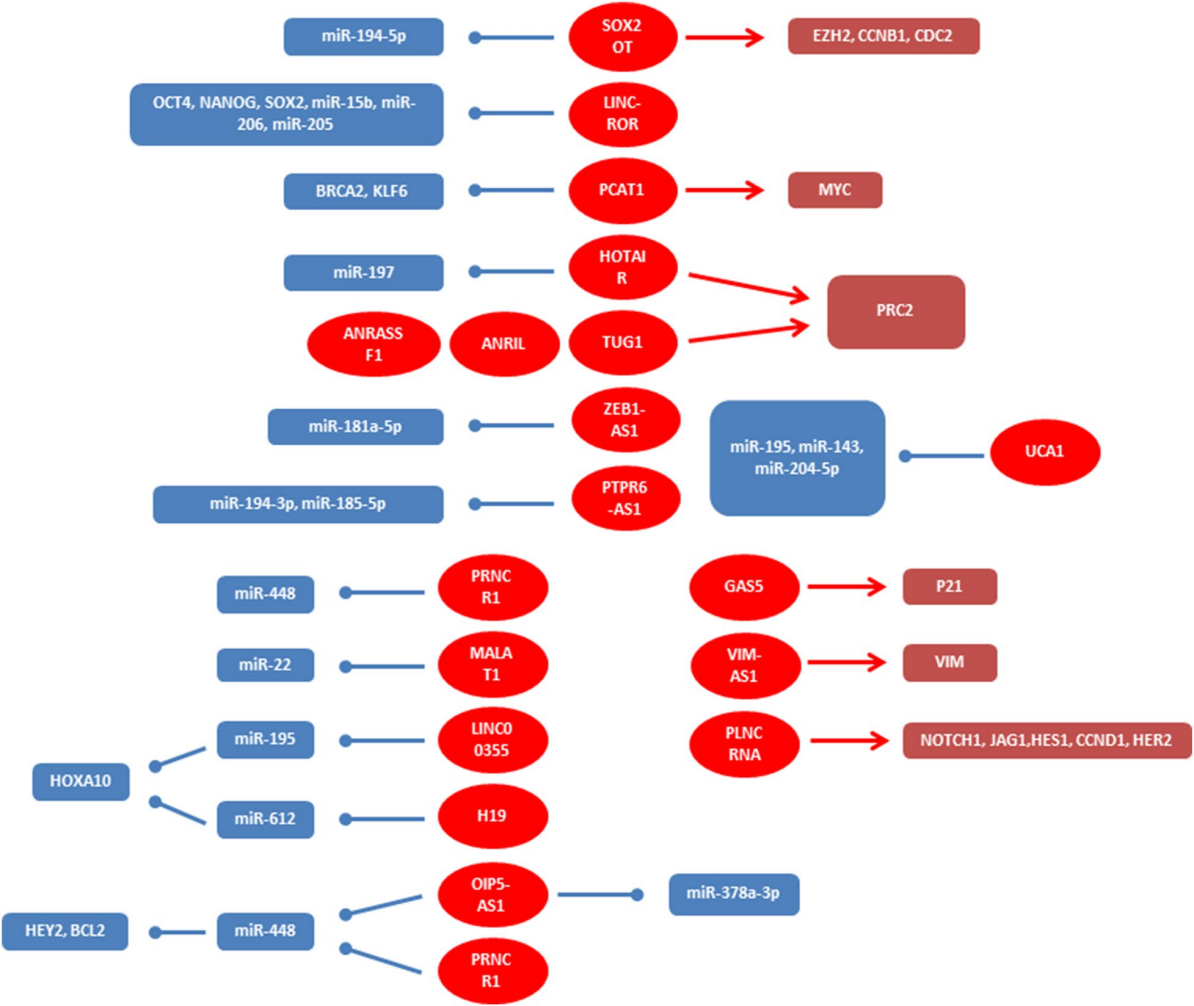
Molecular mechanisms and target genes of lnRNAs

Cancer is one of the main global health challenges and is the leading and second causes of deaths among developed and developing nations, respectively [1]. Cancer is the third common cause of deaths after heart disorders and accidents in Iran with mortality rates of 65 and 41.1 per 100,000 among men and women [2, 3]. Industrialization, rapid development, and lifestyle changes affect the epidemiological patterns of malignancies among Iranians [4, 5]. There are various genetic and environmental factors associated with cancer. The common global environmental risk factors are smoking, alcohol consumption, diet, and lack of physical activity. The most important risk factor for cancer is tobacco that involves about 22% of cancer deaths [6]. Moreover, some chronic infections including Human papillomavirus (HPV), Hepatitis B virus (HBV), Hepatitis C virus (HCV), Epstein–Barr virus (EBV), and Helicobacter pylori are also contributed with cancer, particularly in less developed countries [7]. Smoking and overweight are the most important environmental risk factors for lung and gastric cancers in Iranian subjects [8, 9]. Beside the environmental factors, genetic and epigenetic factors are also involved in cancer susceptibility among Iranians [10–12]. Family history has been reported as a risk factor for breast cancer among Iranian patients [13]. Epigenetic mechanisms such as DNA methylation and RNA-associated silencing are critical processes during normal development and tumor progression. Epigenetic disruption can change the function of key pathways in malignant cells [14, 15]. Although, majority of human genome are non-coding sequences, most of diagnostic and therapeutic markers are based on coding sequences. Up to 90% of non-coding sequences are transcribed to the non-coding RNAs. Noncoding RNAs (ncRNAs) comprise more than 90% of the human genome which are classified into small ncRNAs (< 200 bp) and Long non coding RNAs (lncRNAs) (> 200 bp). They play important roles in epigenetic modifications, transcriptional/Post-transcriptional regulation, and transposon control [16]. LncRNAs regulate gene expression or chromosome activity through DNA methylation, histone modification, and RNA interaction [17]. They are also classified into lncRNA, Long-intergenic noncoding RNA (lincRNA), very long intergenic noncoding RNA (vlincRNA), macroRNA, and Promoter-associated long RNA (PALR). LncRNAs have critical roles in regulation of various biological processes such as immune response, imprinting, and alternative splicing. Deregulation of lncRNA is associated with tumor progression [18]. The majority of somatic mutations, copy number variations and cancer related SNPs are associated with non-coding RNAs [19, 20]. SNPs of lncRNAs have been reported in various cancers. Moreover, deregulation of lncRNA targets are associated with stage, prognosis, and drug resistance [21–23]. Since there is a raising trend of cancer incidence during recent years, it is required to introduce novel population based diagnostic panel markers for the early detection of cancers among Iranians. Early detection is a critical issue in cancer treatment which is commonly done by various methods such as magnetic resonance imaging (MRI), histopathology, and molecular pathology. However, none or minimally invasive methods are required. High stability in body fluids, exosomes, and apoptotic bodies make the lncRNAs as reliable diagnostic and prognostic biomarkers in cancer [24]. Moreover, deregulated pattern of lncRNAs in tumor tissues is mirrored in body fluids such as blood, urine, and saliva [25–27]. Therefore, lncRNA-based biomarkers are low invasive compared with common biopsies [28]. In contrast with mRNA, the lncRNA is itself functional and its expression level can be a better diagnostic marker. Moreover, the specific expression patterns of lncRNAs introduce their expression signatures for the diagnosis and disease classification. However, lncRNAs are transcribed at lower rates than that of mRNAs which makes lncRNA detection as less sensitive methods. Gene therapy also targets the harmful lncRNAs or delivers beneficial lncRNAs such as tumor suppressor or drug sensitive lncRNAs to specific cells [29]. Although, both of miRNAs and lncRNAs are stable in body fluids and can be used as the markers for some diseases, lncRNAs exert their biological function as endogenous decoys for miRNAs. Therefore, the lnRNAs can be suggested as upstream regulators of miRNAs and mainly exert their inhibitory role on other proteins through miRNAs mediators (Fig. 1). The available cancer drugs cannot eliminate the tumor cells completely that results in tumor relapse. Therefore, various lncRNAs such as H19 and HOTAIR can also be used as markers of tumor recurrence [30, 31]. lncRNAs can also be associated with epigenetic machinery. HOTAIR forms a repressor complex through interaction with PRC2 and LSD1 that leads in tumor invasion [32]. Moreover, lncRNAs regulate the epithelial-to-mesenchymal transition (EMT) process. Zeb2 NAT prevents ZEB2 splicing which suppresses CDH1 [33].

**Fig. 2:**
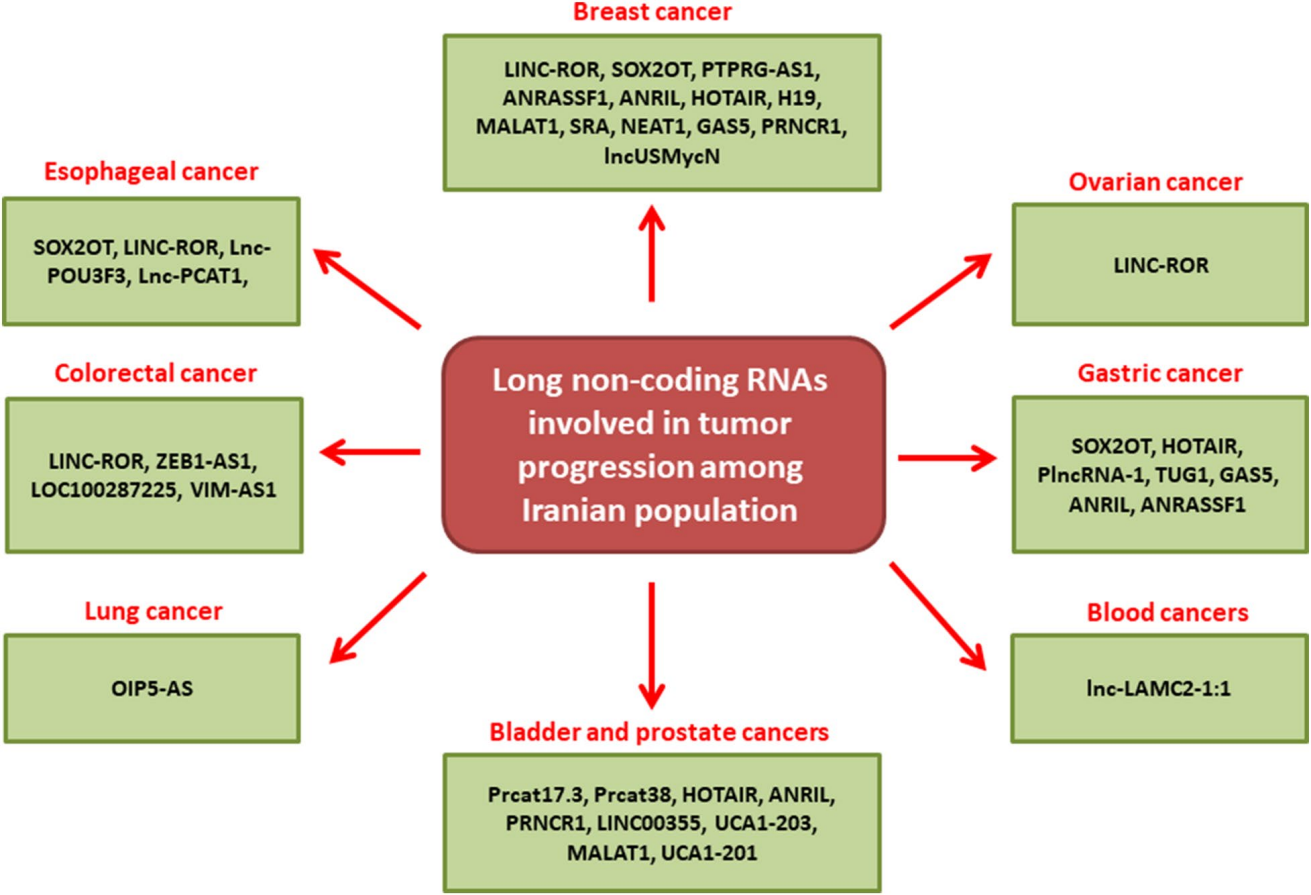
All of the long noncoding RNAs which are involved in tumor progression among Iranian population

In present review we have summarized all of the lncRNAs with significant roles during tumor progression which have been reported until now among Iranian cancer patients to pave the way of introducing a non-invasive population based diagnostic panel of lncRNAs (Table 1). We categorized all of the reported lncRNAs based on tumor types among Iranian population (Fig. 2). Moreover, all of the molecular mechanisms and target genes of reported lncRNAs are illustrated in (Fig. 1).

**Table 1:** All of the long non coding RNAs involved in tumor progressions among Iranian patients

Study (et al.)	Year	Type	Gene	Population	Results
Shahryari [37]	2014	ESCC	SOX2OT	36 N/T^a^	Over expression
Rezaei [45]	2016	Esophageal, ovarian, cervical, breast, sarcoma, colon, and melanoma	LINC-ROR	27 N/T	Esophageal, ovarian, and cervical cancers had over expressions while breast, sarcoma, colon, and melanoma had under expressions
Sahebi [49]	2016	ESCC	LINC-ROR	30 N/T	Over expression
Rahimnia [51]	2018	ESCC	lnc-POU3F3	32 patients 32 controls	Over expression
Razavi [56]	2019	ESCC	lnc-PCAT-1	75 N/T	Over expression
Bayat [58]	2018	Prostate	Prcat17.3, Prcat38, Cat2184.4	30 N/T	Prcat17.3 and Prcat38 over expressions and Cat2184.4 under expression
Taheri [60]	2017	Prostate	HOTAIR	128 patients 250 controls	Polymorphism was correlated with tumor progression
Taheri [64]	2017	Prostate	ANRIL	125 patients 220 controls	Polymorphism was correlated with tumor progression
Sattarifard [66]	2017	Prostate	PRNCR1	178 patients 180 BPH^b^	Polymorphism was correlated with tumor progression
Yazarlou [83]	2018	Bladder	LINC00355, UCA1-203, MALAT1, and UCA1-201	59 patients 24 controls	LINC00355, UCA1-203, and MALAT1 over expressions. UCA1-201 under expression
Farhangian [88]	2018	Gastric	SOX2OT	33 N/T	Under expression
Hajjari [90]	2013	Gastric	HOTAIR	31 N/T	Over expression
Emadi-Andani [91]	2014	Gastric	HOTAIR	60 N/T	Over expression
Baratieh [97]	2017	Gastric	PlncRNA-1 and TUG1	70 N/T	Over expression
Aminian [103]	2019	Gastric	GAS5	130 patients 230 controls	Polymorphism was correlated with tumor progression
Kangarlouei [106]	2019	Gastric	ANRIL and ANRASSF1	39 N/T	Over expression
Iranpour [111]	2016	Breast	SOX2OT, PTPRG-AS1, ANRASSF1, and ANRIL	38 N/T	Over expression
Hassanzarei [114]	2017	Breast	HOTAIR	220 patients 231 controls	Polymorphism was correlated with tumor progression
Hassanzarei [118]	2017	Breast	H19	230 patients 240 controls	Polymorphism was correlated with tumor progression
Safari [119]	2019	Breast	H19	111 patients 130 controls	Polymorphism was correlated with tumor progression
Abdollahzadeh [120]	2019	Breast	H19	150 patients 100 controls	Polymorphism was correlated with tumor progression
Arshi [130]	2018	Breast	MALAT1, SRA, NEAT1, and GAS5	23 patients 15 controls	MALAT1, SRA, and NEAT1 over expressions. GAS5 under expression
Soleimanpour [135]	2018	Breast	PRNCR1	30 N/T	Over expression
Ravanbakhsh [138]	2019	Breast	lncUSMycN	52 N/T	Over expression
Rezanejad Bardaji [140]	2018	Colorectal	ZEB1-AS1	32 N/T	Over expression
Kazemzadeh [143]	2017	Colorectal	LOC100287225	30 N/T	Under expression
Kazemzadeh [142]	2016	Colorectal	LOC100287225	39 N/T	Under expression
Rezanejad bardaji [144]	2018	Colorectal	VIM-AS1	35 N/T	Over expression
Hashemi [146]	2016	ALL	lnc-LAMC2-1:1	110 patients 120 controls	Polymorphism was correlated with tumor progression
Esfandi [150]	2018	Lung	OIP5-AS	32 N/T	Under expression

^a^Tumor tissues and normal margins

^b^Benign prostatic hyperplasia

## Main text

### Esophageal cancer

SOX2 overlapping transcript (SOX2OT) is an lncRNA located on 3q26.33. The SOX2 is located within the third intron of SOX2OT gene which is transcribed in the same orientation. There are 13 splice variants for SOX2OT [34]. SOX2OT plays a key role in regulation of SOX2 expression which has been implicated in various human cancer tissues [35]. Moreover, SOX2OT is involved in regulation of chromatin structure and transcription [34]. SOX2OT functions as the competitive endogenous RNAs (ceRNAs) in which it plays as a sponge LncRNA of miR-194-5p to regulate the AKT2 in GC [36]. It has been observed that there were significant increased levels of SOX2OT, SOX2OT-S1, and SOX2OT-S2 expressions in tumors compared with normal margins among a subpopulation of Iranian Esophageal Squamous Cell Carcinoma (ESCC) patients [37]. Similarly, it has been reported that the SOX2OT and SOX2 were significantly increased in a sample of Chinese ESCC tissues compared with normal samples. Moreover, SOX2 and SOX2OT expression levels were correlated with stage of tumor. They also showed that the SOX2OT expression was directly correlated with SOX2 expression in tumor tissues [38].

LincRNA-Regulator of Reprogramming (lincRNA-RoR) regulates the reprogramming process in pluripotent stem cells. Moreover, it is associated with iPSC derivation and ESC pluripotency [39]. Aberrant LINC-ROR expression can be related with cell proliferation, invasion, hypoxic response, and tumor progression [40]. Linc-ROR has an important role during DNA damage response. ROR inhibits the P53 translation via heterogeneous nuclear ribonucleoprotein I (hnRNPI) and RNA-binding protein which play critical role in splicing [41]. Moreover, linc-ROR is involved in regulation of histone modifications and cellular responses to chemotherapy [42, 43]. LINC-ROR acts as a molecular sponge for the core transcription factors such as OCT4, NANOG, and SOX2 [44]. It has been reported that there were a heterogeneous pattern of LINC-ROR expression among Iranian patients in which the esophageal, ovarian, and cervical cancers had over expressions, while there were LINC-ROR down regulations in breast, sarcoma, colon, and melanoma cancer cases [45]. Endogenous linc-ROR acts as a ceRNA of SOX9 during ESCC progression through sponging multiple miRNAs such as miR-15b and miR-206 [46]. Moreover, linc-ROR functions as a sponge for miR-205 to regulate EMT which is known to have a vital role in diverse physiological and pathological processes [47]. It has been found that the mutual inhibition mechanism between ROR and miR-133 mediates the reprogramming in cardiac hypertrophy [48]. It has been reported that the linc-ROR and its variants 2 and 4 were significantly up-regulated in a sample of Iranian ESCC patients compared with normal margins. There was significant association between variant 4 expression level and tumor grade [49]. Similarly, there were significant LincRNA ROR upregulations in ESCC tissue samples compared with adjacent normal tissues in Chinese ESCC patients. Moreover, they showed significant correlations between LincRNA ROR expression levels, lymph node and distant metastasis, and TNM staging [50].

Lnc-POU3F3 is located on the reverse strand of chromosome 2q12.1 and POU3F3 upstream which is a member of the class III POU family of transcription factors [51]. POU3F3 over expression has been reported in ESCC cases [52]. It has been observed that there were significant higher levels of lnc-POU3F3 in blood samples among a sub population of Iranian ESCC patients compared with healthy subjects. There were also significant correlation between lnc-POU3F3, family history, and TNM stage [51]. Similarly, there was significant increased plasma levels of POU3F3 in Chinese ESCC patients compared with normal controls [52]. Another group also reported the linc-POU3F3 up regulation in Chinese colorectal cancer patients which was associated with tumor grade and stage [53].

Prostate cancer associated transcript 1 (PCAT1) acts as an oncogene through inhibition of BRCA2 and stimulation of MYC. It promotes tumor cell invasion through targeting RBM5 and KLF6 in pancreatic and ovarian cancer [54, 55]. It has been indicated that there were significant increased levels of lnc-PCAT-1 in a group of Iranian ESCC samples compared with normal margins. Moreover, the results showed a significant correlation between up-regulating of lncRNA- PCAT-1 and hot liquid drinking [56]. Similarly, it has been reported that the lncRNA PCAT-1 was significantly overexpressed in ESCC compared with the adjacent noncancerous tissues in Chinese patients. Moreover, high levels of PCAT-1 expressions were significantly associated with invasion, advanced stage, lymph node involvement, and poor prognosis [57].

### Prostate and bladder cancers

Prcat17.3 and Prcat38 are located upstream of TMPRSS2 gene, which are used to distinguish malignant and nonmalignant prostate tissues. Prcat17.3 and Prcat38 are positively correlated with TMPRSS2 in prostate cancer. Cat2184.4 is located upstream of PMEPA1 which acts as a tumor suppressor in prostate tumorigenesis. It has been shown that there were significant up regulations of Prcat17.3 and Prcat38 and significant down regulation of Cat2184.4 in prostate cancer (PCa) tissues compared with benign prostate hyperplasia (BPH) among a group of Iranian subjects. Moreover, the results showed a significant up regulation of Prcat17.3 level in urine samples of PCa patients compared with BPH patients. According to the ROC curve analysis, it has been demonstrated that the Prcat17.3 urine assay has a better sensitivity in distinguishing PCa from BPH compared with urine PCA3 tests. Therefore, Prcat17.3 and Prcat38 can be suggested alone or in combination with PCA3 as diagnostic markers of PCa among Iranians [58].

HOTAIR is an oncogenic factor transcribed from the antisense strand of HOXC locus Which is involved in epigenetic regulation through interaction with Polycomb Repressive Complex 2 (PRC2) [59]. HOTAIR expression is regulated through different pathways such as DNA methylation and HOTAIR inhibition through AGO2, miR-141, and Osteopontin [59]. It has been reported that the HOTAIR rs1899663 T allele was associated with BPH risk. The rs12826786 T allele was also significantly correlated with BPH and PCa in codominant and recessive models compared with healthy subjects [60]. Regarding the rs12826786, the CC genotype was significantly correlated with shorter survival in Portuguese pT3-stage PCa cases [61].

ANRIL is an lnc-RNA which binds with CBX7 and SUZ12 within the PRC1 and PRC2 to regulate the transcriptional repression [62, 63]. It has been demonstrated that the rs4977574, rs1333048, and rs10757278 genotypes of ANRIL were significantly correlated with PCa and BPH risk among Iranian subjects [64]. Similarly, it has been observed that there was a correlation between ANRIL and PCa progression among Chinese cases, in which the tumors had higher levels of ANRIL expressions compared with normal margins. Moreover, they showed that the ANRIL silencing inhibited the PCa proliferation and migration in several cell lines which can be related to the TGF-β, SMAD2, let7a, and SMAD7 [65].

Prostate cancer associated non-coding RNA 1 (PRNCR1) is located in 8q24.21 and highly expressed in aggressive PCa. It has a critical role in PCa progression through regulation of androgen receptor (AR) [66]. Besides, the PRNCR1 acts as an oncogene in colorectal cancer [67] and gastric cancer [68]. PRNCR1 acts as a ceRNA by sponging miR-448 to modulate HEY2 [68]. It has been shown that there was a significant correlation between the rs13252298, rs1456315, and rs7841060 genetic polymorphisms of PRNCR1 and increased risk of PCa in a sample of the Iranian population [66]. The PRNCR1 polymorphisms were also associated with gastric cancer risk among Korean and Chinese Populations [69, 70]. Another study also showed a significant correlation between PRNCR1 polymorphisms and up regulation and increased CRC susceptibility in Saudi subjects [71].

Long intergenic non-protein coding RNA 355 (LINC00355) is associated with various cellular processes such as apoptosis, proliferation, and migration [72, 73]. It has been demonstrated that the LINC00355 is up regulated in bladder cancer [72]. LINC00355 expression changes significantly are associated with pathological stages of colorectal cancer [73]. MALAT1 has critical roles in nuclear organization, epigenetic regulation, cell migration, and tumor progression [74–76]. MALAT1 is involved in alternative splicing through interaction with several SR splicing factors including SRSF1, 2, and 3 [77]. MALAT1 expression level is also involved in cell cycle progression and invasion through the regulation of apoptotic genes expression including CASP3, CASP8, BAX, BCL-2, and BCL-XL [78]. MALAT1 acts as a competing endogenous RNA (ceRNA) by sponging miR-22 and regulation of MMP14 and SNAIL [79]. Urothelial cancer associated 1 (UCA1) is involved in regulation of mitochondrial metabolism through miR-195/ARL2 pathway in bladder cancer [80]. It promotes EMT process in bladder tumor cells via modulation of miR-143/HMGB1 pathway [81]. Moreover, it has been shown that the UCA1 increases drug resistance through WNT signaling pathway in bladder cancer [82]. It has been reported that there were up regulations of LINC00355, UCA1-203, and MALAT1 and down regulation of UCA1-201 in urinary exosomes isolated from transitional cell carcinoma (TCC) of bladder compared with controls in Iranian patients. Moreover, they introduced (UCA1-201, UCA1-203, MALAT1, and LINC00355) as a diagnostic panel of lncRNAs in bladder cancer [83]. Similarly, it has been reported that there was MALAT1 over expression in a sample of Chinese bladder cancer patients compared with normal margins. They showed associations between the levels of MALAT1 expression and clinicopathological features including advanced grade, high tumor stage, and lymph node involvement [84]. Similar to the results among Iranian bladder cancer, it has been reported that the UCA1 expression was significantly up-regulated in CRC tissues compared with normal margins. Moreover, they showed that the UCA1 acts as a ceRNA by sponging miR-204-5p and regulation of CREB1 which resulted in 5-FU resistance in CRC patients [85].

### Gastric cancer

SOX2OT is an lncRNA that is de-regulated in tumor tissues and is down regulated during the cell differentiation [34, 37]. Moreover, it regulates the cell cycle through EZH2 [86]. It has been reported that the SOX2OT was significantly down regulated in tumors compared with normal gastric samples in a sample of Iranian subjects. There were also decreased expression levels in high grade compared with low grade tumors [87]. Similarly, it was shown that the SOX2OT expression levels were significantly lower in cancerous tissues compared with normal margins in Chinese cases. Moreover, SOX2OT expression was correlated with distant metastasis and differentiation. The findings demonstrated that the SOX2OT over expression was associated with aggressive tumor behavior [88]. In contrast with the Iranian GC patients, there was a significant SOX2OT up regulation in a sample of Chinese gastric tumors compared with normal which was associated with poor prognosis and invasive status [89].

The findings demonstrated that the relative expression of HOTAIR long non-coding RNA was significantly up regulated in GC tissues compared with normal margins in a sample of Iranian subjects. There was also association between HOTAIR expression level with TNM staging and lymph node metastasis. Moreover, they showed a significant direct association between the levels of HOTAIR and SUZ12 expressions [90]. Another study has been also observed that the HOTAIR expression levels were significantly elevated in a sample of Iranian GC tissues compared with normal margins which was associated with TNM staging, perineural invasion, and distant metastasis [91]. Similarly, HOTAIR was significantly up regulated in Chinese GC tissues. It has been revealed that the HOTAIR expression was positively associated with tumor differentiation, lymph node involvement, and clinical stage [92]. HOTAIR expression was significantly higher in Chinese CRC tissues than in normal tissues. It may promotes the CRC progression through sponging miR-197 [93]. Another study on Chinese CRC cases showed significant over expression of HOTAIR in tumor compared with normal margins. Moreover, HOTAIR expression was significantly associated with lymph node metastasis, differentiation, and clinical stage [94].

TUG1 plays a critical role in epigenetic regulation through interaction with PRC2 or PRC1 [95]. It induces cell proliferation and acts as a miR sponge. It has been reported that the TUG1 plays a role in promotion of cell growth and drug resistance in small cell lung cancer (SCLC) via regulation of LIMK2b by EZH2 binding [96]. PlncRNA-1 is encoded from the antisense strand of the carbonyl reductase 3 (CBR3) on chromosome 21q. PlncRNA-1 is over expressed in various cancers including PCa, ESCC, and hepatocellular carcinoma (HCC) [97]. It has been revealed that there were significant higher levels of PlncRNA-1 and TUG1 expressions in a sub population of Iranian GC tissues compared with normal margins. The PlncRNA-1 expression level was correlated with sex in which female patients had a significantly PlncRNA-1 over expression compared with males [97]. PlncRNA-1 expression level was significantly over expressed in Chinese HCC tissues that was significantly associated with tumor size and advanced TNM stage [98]. Similar to the results among Iranian population, TUG1 expression level was significantly increased and associated with GC outcomes in Chinese cases. Moreover, there were significant associations between TUG1 levels and clinicopathological features including tumor depth of invasion and advanced TNM stage [99].

Growth arrest specific 5 (GAS5) is an ncRNA that inhibits the glucocorticoid receptor via binding with its DNA binding domain. It regulates gastric cell proliferation, apoptosis, and migration through CDK6, YBX1, and P53 [100–102]. It has been reported that there was a significant correlation between the del allele of rs145204276 polymorphism of GAS5 lncRNA and decreased risk of GC in a sample of Iranian subjects [103]. Similarly, GAS5 rs145204276 polymorphism was a functional variant associated with the risk and metastasis of GC in a group of Chinese patients [104]. GAS5 was also protective in glioma in which there was reduced expression levels of GAS5 in glioma compared with normal brain tissues. Moreover, GAS5 expression was negatively associated with the tumor grades [105].

ANRASSF1 is an antisense lncRNA of RASSF1 involved in epigenetic regulation through binding with PRC2 that is required for recruiting PRC2 to the RASSF1A promoter region. This process results in accumulation of the H3K27me3 and decreases the RASSF1A protein. It has been observed that there were significant increased levels of ANRIL and ANRASSF1 in GC tumors compared with the normal margins among a subpopulation of Iranian patients [106]. ANRIL was also up regulated in human osteosarcoma tissues. Moreover, it was shown that the ANRIL was associated with tumor cell growth, apoptosis, and migration through CASP3, BCL2, and CDH1 in osteosarcoma [107].

### Breast cancer

Protein tyrosine phosphatase receptor type G antisense (PTPRG-AS1) is an antisense lncRNA of PTPRG which has important roles in cell growth, differentiation, and neoplastic transformation. PTPRG-AS1 is correlated with ER+ and ER −  subtypes, tumor grade, and clinical results [108]. It functions as a sponge of miR-194-3p to regulate the radio resistance and invasiveness in nasopharyngeal tumor cells through PRC1 [109]. ANRIL is an oncogenic lncRNA, which has a regulatory epigenetic role on p15/CDKN2B-p16/CDKN2A-p14/ARF via recruitment of PRC2 and PRC1 [110]. It has been reported that there were significant increased expression levels of SOX2OT, PTPRG-AS1, ANRASSF1, and ANRIL in Iranian breast cancer tissues compared with normal margins. Moreover, a significant association was observed between ANRASSF1 expression level and Her2/neu negative status. ANRASSF1 and ANRIL expressions were also significantly up regulated in triple negative cases [111]. Similarly, ANRIL over expression was reported in a large cohort of invasive French breast cancer patients compared with normal breast tissues [112]. Similar to the results in Iranian breast cancer cases, PTPRG-AS1 over expression has been reported in Chinese glioma samples compared with matched adjacent normal tissues. Moreover, they showed that the PTPRG-AS1 acts as a ceRNA by sponging miR-185-5p to regulate cell growth [113].

It has been reported that there were a significant association between HOTAIR polymorphisms and risk of breast cancer in a sample of southeast Iranian population in which rs920778 polymorphism significantly increased breast cancer risk while the rs12826786 and rs1899663 polymorphisms significantly decreased breast cancer risk. Moreover, rs920778 and rs12826786 were significantly correlated with ER status [114]. Similarly, it has been observed that there was a correlation between the CC genotype of HOTAIR rs920778 and clinicopathological features such as advanced stage, distant metastasis, and poorly differentiation among a Turkish breast cancer population [115].

H19 is a paternally imprinted gene associated with the human Beckwith-Wiedemann syndrome that is located on chromosome 11p15.5. H19 acts as an oncogene in bladder cancer, breast cancer, and hepatocellular carcinoma. It is associated with E2F1 transcription factor to induce breast cancer cell cycle progression [116]. H19 is up regulated in extra embryonic tissues and most fetal tissues but its expression is dramatically decreased after birth. The lncRNA-H19 plays important role in mesenchymal stem cells proliferation and regulation of their lineage differentiation [117]. It has been observed that the rs3741219, rs217727, and rs2839698 polymorphisms of H19 were significantly associated with increased risk of breast cancer, while the rs3741216 was significantly contributed with reduced risk of BC among a southeast sub population of Iranian patients [118]. Another study on Iranian breast cancer cases showed that the T allele of rs2839698 and T allele of rs217727 had susceptible and protective effects respectively [119]. The role of rs217727 C>T polymorphism in breast cancer pathogenesis was also confirmed by another group among Iranian patients [120]. Similarly, rs217727 T variant of H19 was significantly correlated with increased risk of breast cancer among Chinese cases [121]. Other polymorphisms of H19 such as rs4930101, rs11042170, and rs27359703 significantly increased the risk of Chinese colorectal cancer patients [122]. Li et al. also reported that the H19 rs217727 SNP was associated with the risk of lung cancer in a Chinese population [123]. The lncRNA H19 was overexpressed in a sample of French breast adenocarcinomas compared with healthy tissues which was also significantly associated with the presence of both estrogen and progesterone receptors [124].

Steroid receptor RNA activator (SRA) regulates gene expression by steroid hormones. SRA is involved in regulation of physiological processes such as NR signaling, steroidogenesis, and mesenchymal fate. SRA functions as a molecular scaffold to regulate the transcription by various co-regulators and chromatin-modifying factors in both activating and repressive complexes [125]. GAS5 is up regulated in growth arrested cells, and functions as a hormone response element for the glucocorticoid receptor (GR) [126]. GAS5 is associated with apoptosis and risk of ischemic stroke [127]. Moreover, GAS5 acts as a tumor suppressor in glioma [105]. NEAT1 is a single-exon lincRNA and interacts with P54nrb or NONO which are necessary for the formation of nuclear paraspeckles in the inter chromatin space [128]. NEAT1 also regulates the expression of some chemokines and cytokines such as IL-6 and CXCL10 through the MAPK signaling pathway [129]. It has been revealed that there were significant up regulations of MALAT1, SRA, and NEAT1, while significant GAS5 down regulation in Iranian breast cancer samples which were taken from younger (< 45 years) and older (> 45 years) cases compared with normal tissues [130]. MALAT1 expression level was significantly up regulated in breast tumor tissues than adjacent non-cancerous tissues among Chinese population. Moreover, they showed that the MALAT1 promoted angiogenesis which can be associated with miR-145 [131]. It has been also observed that the GAS5 were down regulated in Chinese Triple-negative breast cancer (TNBC) patients which was associated with tumor aggressiveness, lymph node metastasis, and survival [132]. Yan et al. showed that the SRA rs10463297 TC polymorphism significantly increased the risk of breast cancer compared with CC genotype in a sample of Chinese subjects [133]. Similar to the results in Iranian breast cancer patients, Shin et al. showed that the NEAT1 expression was increased in peripheral blood of breast cancer patients compared with normal controls in Chinese cases. Moreover, NEAT1 expression was more prominent in TNBC tissues than other subtypes (ductal carcinoma in situ, luminal, HER2, and TNBC) [134].

PRNCR1 is a critical AR regulator. It has been reported that there were significant increased levels of PRNCR1 expressions in breast tumor tissues compared with normal margins among a sub population of Iranian subjects. Moreover, there were significant association between PRNCR1 overexpression and clinical-pathological features such as tumor size and lymph node involvement [135]. Similarly, another study has been also reported that there were significant increased expressions of PRNCR1 in the plasma of breast cancer patients compared with healthy cases among a sub population of Iranian cases [136].

The NAV2-AS2 is a lncRNA located in 11p15.1 and the minus strand of NAV2 sequence [137]. NAV2 is belonged to the family of neuron navigators which is associated with cell growth, migration, and development. It has been observed that there were significant increased levels of NAV2-AS2 expressions in Iranian breast lobular carcinoma which was associated with a higher risk of lobular carcinoma and disease grade [137].

Long non-coding upstream of MYCN (lncUSMycN) is transcribed from the 14-kbp upstream of the MYCN transcription start site. It has been reported that the lncUSMycN up regulated N-Myc mRNA expression through non O protein. It has also important roles in cell proliferation and tumorigenesis in neuroblastoma. There was significant up regulation of lncUSMycN among a sample of Iranian breast cancer patients compared with healthy controls. Moreover, levels of lncUSMycN expression was significantly associated with the early stages of breast cancer [138].

### Colorectal cancer

LncRNA ZEB1 antisense 1 (ZEB1-AS1) is transcribed from the ZEB1 promoter sequence and it regulates the ZEB1expression levels. ZEB1-AS1 binds with MLL1 to induce H3K4me3 in ZEB1 promoter sequence. It promotes colorectal tumor cell proliferation through p15 inhibition [139]. It has been shown that there were significant up regulations of ZEB1-AS1 in tumor tissues compared with normal margins in a sub population of Iranian CRC patients. Moreover, the ZEB1- AS1 expression was correlated with tumor stage, lymph node metastasis, and vascular invasion [140]. Similarly, it has been observed that the ZEB1-AS1 expression was significantly up regulated in colorectal cancer compared with adjacent normal tissues among Chinese cases. The patients with high expression level of ZEB1-AS1 had poorer overall survival in comparison with the ZEB1-AS1 down regulated patients [139]. Another group also reported the ZEB1-AS1 over expression among Chinese colorectal cancer cases. Moreover, they showed that the ZEB1-AS1 promotes tumor cell proliferation via regulation of WNT signaling pathway and miR-181a-5p sponging [141].

LOC100287225 is an lncRNAs located in the long arm of the chromosome 18, upstream region of deleted in colorectal carcinoma (DCC). DCC is a trans-membrane cell adhesion protein belonging to the immunoglobulin family which is associated with axon attraction and apoptosis induction. It has been demonstrated that there were significant LOC100287225 down regulations in tumor tissues compared with adjacent tumor-free tissue among a sample of Iranian CRC patients [142, 143].

VIM-AS1 RNA is located in 10p13 that is transcribed from a shared bidirectional promoter with Vimentin mRNA [144]. It has been observed that the VIM-AS1 was significantly over expressed in tumors with high-grade, lymph node metastasis, and vascular invasion among a sub population of Iranian CRC patients. The VIM-AS1 down regulation could also suppress tumor cell proliferation through apoptosis induction and cell cycle inhibition. Moreover, VIM-AS1 had a critical role in epithelial to mesenchymal transition (EMT) of colorectal tumor cells. They introduced the VIM-AS1 as a probable diagnostic marker of CRC among Iranians [144].

### Leukemia and lung cancer

Lnc-LAMC2-1:1 is located in 1q25.3 and overlaps with LAMC1. It has been reported the genetic variation in lnc-LAMC2-1:1 was associated with CRC by affecting miRNA binding [145]. The lnc-LAMC2-1:1 rs2147578 C>G polymorphism was significantly correlated with childhood ALL progression in a sample of Iranian population. Moreover, there was a significant association between rs2147578 C>G and platelet count [146]. Another study showed that the CG and GG genotypes of the rs2147578 were significantly correlated with higher risk of CRC in comparison with the rs2147578 CC genotype among Chinese subjects. Moreover, they showed that the rs2147578 influences the lnc-LAMC2-1:1/miR-128-3p interaction [145].

OIP5-AS1 functions as a sponge for miR-448 to regulates BCL-2 in lung adenocarcinoma cells [147]. OIP5-AS1 acts as a sponge for HuR to decrease its activity to target CCNA2, CCND1, and SIRT1 mRNAs [148]. Moreover, OIP5-AS1 functions as a ceRNA for miR-195-5p to up regulate NOB1 in human hemangioma endothelial cells [149]. It has been observed that there were significant decreased levels of OIP5-AS among a sub population of Iranian non-small cell lung cancer (NSCLC) samples compared with their corresponding normal margins [150]. In contrast with the results among Iranian NSCLC cases, it has been observed that there was OPI5-AS1 up regulation in a sample of Chinese lung adenocarcinoma tissues. Moreover, the OIP5-AS1 expression levels were associated with poor prognosis and clinical grade in lung adenocarcinoma patients [147]. Another study in Chinese population also showed OIP5-AS1 up regulation in lung cancer samples which was associated with tumor size and growth. Moreover, it was shown that the OIP5-AS1 promotes lung tumor cell growth via miR-378a-3p inhibition [151].

## Conclusion

There are various diagnostic methods for the early detection of cancer. However, majority of routine methods are invasive. The deregulated pattern of lncRNAs in tumor tissues is mirrored in body fluids which make these factors as a non-invasive candidate with better tolerance for the cancer patients compared with biopsy. Therefore, this review summarized all of the lncRNAs with significant roles during tumor progression which have been reported until now among Iranian cancer patients. This review paves the way of introducing a population based non-invasive panel of lncRNAs for the early detection of cancers among Iranian population.

## Data Availability

The datasets used and/or analyzed during the current study are available from the corresponding author on reasonable request.

## Abbreviations

lncRNAs: long non-coding RNAs
HPV: human papillomavirus
HBV: hepatitis B virus
HCV: hepatitis C virus
EBV: Epstein–Barr virus
ncRNAs: noncoding RNAs
lincRNA: long-intergenic noncoding RNA
vlincRNA: very long intergenic noncoding RNA
PALR: promoter-associated long RNA
SOX2OT: SOX2 overlapping transcript
ceRNAs: competitive endogenous RNAs
ESCC: esophageal squamous cell carcinoma
lincRNA-RoR: lincRNA-regulator of reprogramming
hnRNPI: heterogeneous nuclear ribonucleoprotein I
PCAT1: prostate cancer associated transcript 1
PCa: prostate cancer
BPH: benign prostate hyperplasia
PRC2: polycomb repressive complex 2
PRNCR1: prostate cancer associated non-coding RNA 1
AR: androgen receptor
LINC00355: long intergenic non-protein coding RNA 355
ceRNA: competing endogenous RNA
UCA1: urothelial cancer associated 1
TCC: transitional cell carcinoma
SCLC: small cell lung cancer
CBR3: carbonyl reductase 3
GAS5: growth arrest specific 5
PTPRG-AS1: protein tyrosine phosphatase receptor type G antisense
SRA: steroid receptor RNA activator
GR: glucocorticoid receptor
TNBC: triple-negative breast cancer
lncUSMycN: long non-coding upstream of MYCN
ZEB1-AS1: lncRNA ZEB1 antisense 1
NSCLC: non-small cell lung cancer
